# A System Utilizing Metal Hydride Actuators to Achieve Passive Motion of Toe Joints for Prevention of Pressure Ulcers: A Pilot Study

**DOI:** 10.1155/2012/541383

**Published:** 2012-04-29

**Authors:** Minako Hosono, Shuichi Ino, Mitsuru Sato, Kazuhiko Yamashita, Takashi Izumi

**Affiliations:** ^1^Department of Mechano-Informatics, Graduate School of Information Science and Technology, University of Tokyo, 7-3-1 Hongo, Bunkyo, Tokyo 113-8656, Japan; ^2^Human Technology Research Institute, National Institute of Advanced Industrial Science and Technology, Central 6, 1-1-1 Higashi, Tsukuba, Ibaraki 305-8566, Japan; ^3^School of Nursing and Rehabilitation Science, Showa University, 1865 Tokaichiba, Midori, Yokohama, Kanagawa 226-8555, Japan; ^4^Division of Healthcare Informatics, Faculty of Healthcare, Tokyo Healthcare University, 3-11-3 Setagaya, Setagaya, Tokyo 154-8568, Japan; ^5^Department of Human Science and Informatics, School of Biological Science and Engineering, Tokai University, 1-1-1 Minamisawagojo, Minami, Sapporo, Hokkaido 005-8601, Japan

## Abstract

This paper describes the influence of human toe movement on blood flow and the design of a toe joint passive motion system for preventing pressure ulcers. First, we measured lower extremity blood flow in the foot during active and passive motion of the toe to facilitate the design of new rehabilitation equipment. Also, the flexion and extension angles and the force of the toe joints were measured to determine appropriate specifications for the system. Increases in blood flow were observed at the external malleolus during movement. Flexion and extension angles and the force of the toe joints were found to differ significantly among participants. It is shown that a toe joint passive motion system can be effective in preventing pressure ulcers. On the basis of these results, a device using alloys of metal hydride (MH) as an actuator that is suitable for the system to initiate toe motion, was developed.

## 1. Introduction

Inactivity due to bed rest during convalescence from illness can lead to the development of pressure ulcers. A pressure ulcer is defined as a lesion that is mainly caused by pressure in combination with shear stress. Recent studies indicate that excessive tissue/cell deformation, ischemia, and ischemia-reperfusion due to prolonged loading are important factors in pressure ulcer development [1, 2]. Thus, dispersion of external forces, shortening ischemic period, and increased blood flow at common sites play important roles in the primary prevention or symptomatic improvement of pressure ulcers.

Many researchers have reported on the use of force dispersion to prevent pressure ulcers [3, 4]. Some researchers have developed cushions or mattresses with internal pressure that is controlled by water or air [5, 6], and the thickness, shape, and composition of these cushions have been discussed by other researchers [7–9]. Meanwhile, mechanical stimuli may also be effective in pressure ulcer prevention from microcirculatory point of view [10]. It has been reported that active motion of the human toe or oscillating the lower extremity in an appropriate cycle can increase skin blood flow of the foot [11, 12]. On the basis of these findings, it is expected that toe exercise will have some effect in preventing lower extremity pressure ulcers. However, aggressive exercises for pressure ulcer prevention are difficult to implement when the patient must maintain bed rest. Thus, a preventive exercise or motion system that can be used in a face-up/down position is required.

It is essential for such a system to be simple to use and reliably safe for patients and medical personnel. A system using alloys of metal hydride (MH) as a soft actuator has been developed to meet these needs [13]. MH alloys are capable of hydrogen storage; they can absorb and release hydrogen gas approximately 1,000 times as much as their own volume. By converting this reversible chemical reaction into mechanical power, an MH actuator can generate a high force by a device that is small in size. Additional advantages (e.g., silent operation, zero vibration, and a mechanical buffering action resembling that of a cushion) make an MH actuator suitable as a power source for rehabilitation systems.

In this study, we measured lower extremity blood flow during active and passive motion of the human toe to identify any increases or decreases in blood flow. Then, the flexion and extension angles and the force of the toe joints were measured to facilitate the appropriate design of a new pressure ulcer prevention system. Based on our experimental results, we propose a promising system that applies a soft MH actuator to toe joints for the purpose of preventing pressure ulcers.

## 2. Methods

### 2.1. Participants

Five healthy subjects in their 20s to 50s participated in experiments to measure blood flow during active exercise (experiment 1) and manual passive motion (experiment 2) of their right toe. In addition, ten healthy subjects in their 20s to 80s participated in an experiment to measure blood flow during passive motion of their right toe during use of a continuous passive motion (CPM) device (experiment 3). Ten healthy subjects in their 20s to 60s participated in an experiment to measure the flexion and extension angles and the force of their right toe joints. All participants gave written informed consent. The study was approved by the local institutional review board.

### 2.2. Measurement of Blood Flow in the Human Foot

**Figure 1:**
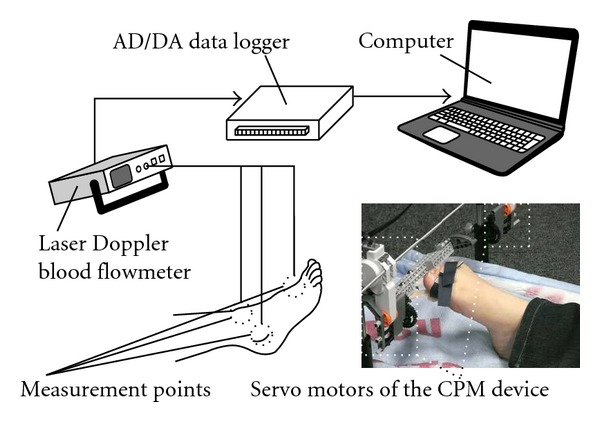
Overview of blood flow measurement.

A measurement of blood flow in the foot was performed to determine whether toe movement caused an increase or decrease in blood flow. An overview of the experiment is shown in Figure 1. Blood flow was measured using a laser Doppler blood flow meter (ALF21, ADVANCE Co., Ltd.), and data were recorded via a USB data logger (USB-6008, National Instruments Co.). The sampling rate was 10 Hz. The locations at which blood flow was measured were the superior surfaces of the first metatarsal head, the external malleolus, and the head of the talus of the right foot as shown in Figure 1. Blood flow was measured from one minute before the start of toe motion to one minute after the end of the motion. All participants were kept at rest in the supine position for five to ten minutes before the measurement and remained in this position until the end of the experiment.

Flexion and extension of the toe were performed alternately through each experiment and considered as one cycle of motion. This cycle was conducted ten times at twelve seconds per cycle during the course of the experiment. Participants executed flexion and extension movements of their toe in the rhythm of a digital metronome in experiment 1. In contrast, an experimenter performed manual flexion and extension of each participant's toe in the rhythm of a digital metronome in experiment 2. A pair of servomotors (LEGO MINDSTORMS NXT, The LEGO Group) was used as part of a CPM device to generate the passive motion applied to each participant's toe in experiment 3, as shown in Figure 1.

### 2.3. Measurement of Flexion and Extension Angles and Force of the Toe Joints

**Figure 2:**
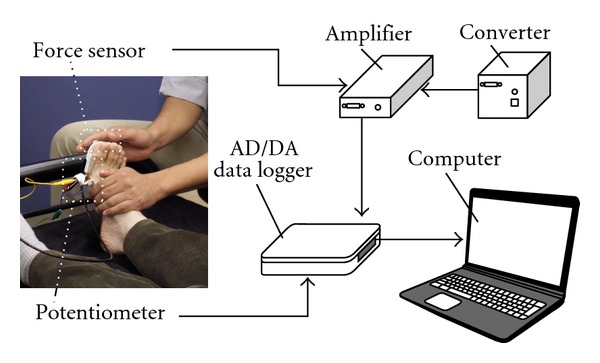
Overview of flexion and extension angles and force measurement of the toe joints.

Flexion and extension angles and the force of the toe joints were measured to determine an appropriate design for a pressure ulcer prevention system. An overview of the experiment is shown in Figure 2. Flexion and extension angles were measured by using a potentiometer (LP06F1F1AA, Murata Manufacturing Co.), and force was measured with a 6-axis force sensor (BL NANO sensor, BL AUTOTEC Ltd.). The data were recorded via a data logger (SCC-68, DAQ card-6024E, National Instruments Co.). The sampling rate was 10 Hz.

 A device was mounted on the foot of each participant to measure the angle of the first metatarsophalangeal (MTP) joint of the right foot (Figure 2). Participants were kept at rest in the supine position during the experiment. A physical therapist flexed and extended each participant's first through fifth toe joints simultaneously utilizing a 6-axis force sensor to measure force (Figure 2). Flexion and extension motions were performed ten times each.

## 3. Results

### 3.1. Changes in Blood Flow of the Foot

**Figure 3:**
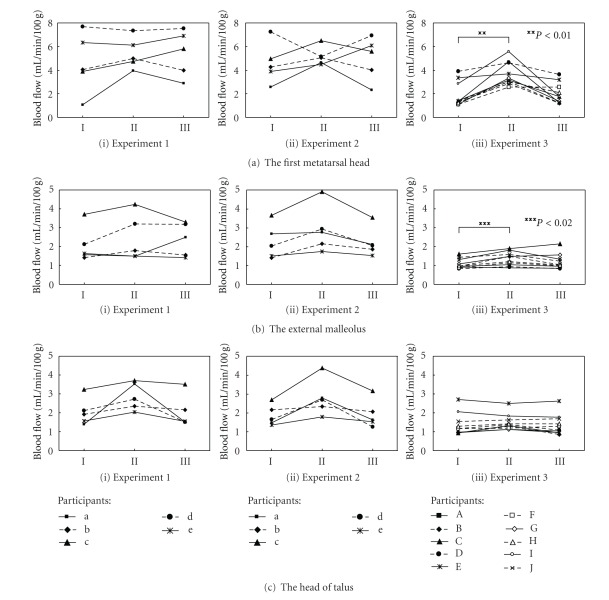
Changes in blood flow of the superior surfaces of (a) the first metatarsal head, (b) the external malleolus, and (c) the head of the talus during (i) active exercise (experiment 1), (ii) manual passive motion (experiment 2), and (iii) passive motion utilizing a CPM device (experiment 3).

Figure 3 shows the changes in the mean value of blood flow at the superior surfaces of the first metatarsal head, the external malleolus, and the head of the talus for each subject in each experiment. In the figure, each participant is represented by a letter of the alphabet. Phases I, II, and III indicate one minute before the motion, two minutes following the start of the motion in each experiment, and one minute after the motion ended, respectively.

 In experiments 1 and 2, there were increases in blood flow at all measurement locations of every participant during phase II or phase III compared with baseline readings recorded during phase I, with the exception of participant d (Figures 3(a)(i) and 3(a)(ii)) and participant e (Figure 3(b)(i)). In experiment 3, increased blood flow at superior surfaces of the first metatarsal head were observed during phase II compared with blood flow measured during phase I (*P* < 0.01, Wilcoxon test) (Figure 3(a)(iii)). The blood flow at the external malleolus (Figure 3(b)(iii)) showed the same increase tendency as observed for the first metatarsal head (*P* < 0.02, Wilcoxon test). Blood flow changes at the head of the talus exhibited no significant differences between phases I and II (Figure 3(c)(iii)).

### 3.2. Flexion and Extension Angles and Force of the Toe Joints

**Figure 4:**
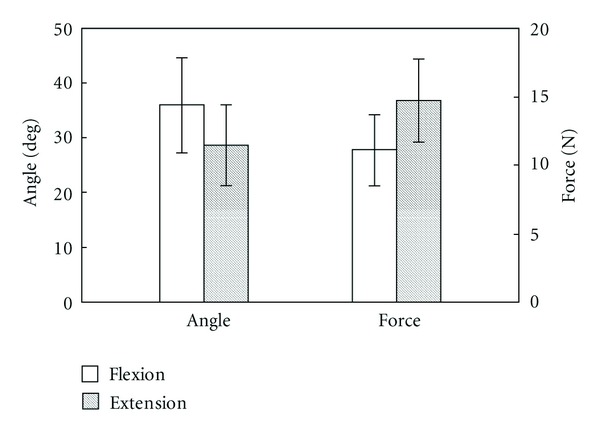
Mean values of the maximum angle of the first MTP joint and the maximum force during flexion and extension. Error bars indicate ±standard deviation.

Mean values of the maximum angles of the MTP joint and the maximum force during flexion and extension are shown in Figure 4. As shown in Figure 4, the maximum force during extension is larger than that during flexion, even though extension angles are smaller than flexion angles. The maximum amount of force during flexion and extension differed among participants, ranging from 8 N to 14 N for flexion and from 9 N to 20 N for extension. Similarly, the maximum angles of the MTP joint differed among participants from 24 degrees to 47 degrees in flexion and from 16 degrees to 40 degrees in extension.

## 4. Discussion

### 4.1. The Utility of a Motion System for Prevention of Pressure Ulcers

No significant differences in blood flow changes were observed at any measurement location between the cases of active exercise (experiment 1) and manual passive motion (experiment 2). Blood flow increases were observed at the superior surfaces of the first metatarsal head and the external malleolus during or after passive motion using a CPM device (experiment 3). The reasons for increased blood flow during passive motion may be a volume change of the blood vessels and/or passive muscle pumping.

 A possible reason for increased blood flow is that blood vessels near the measurement location were forced to deform physically by the motion of toe joints. The superior surface of the first metatarsal head is located closest to the toe joints, and blood flow at that point increased more than blood flow at the external malleolus in experiment 3 (Figures 3(a)(iii) and 3(b)(iii)). At the same time, flexion and extension of the toe joints appeared to induce the muscles of the foot to contract and stretch passively. We believe this led to increased pumping of blood, which influenced blood circulation in the capillary of the foot. We consider that these two factors increased the blood flow as observed at the external malleolus, which is distant from the toe joints, as well as at the superior surface of the first toe. The malleolus is a common site for the development of pressure ulcers [14]. Blood flow increases at the malleolus may, then, be expected to contribute to the prevention of pressure ulcers.

 Two factors discussed above can influence not only microcirculation but also tissue/cell deformation. Recently, the relevance of local excessive deformation toward tissue damage is noted for the etiology of deep tissue injury (DTI) [4, 15, 16]. Linder-Ganz et al. [17] reported the pressure time threshold for cell death of skeletal muscles in rat models. It is hypothesized that the increased levels of intracellular calcium ion caused by sustained tissue deformations correlate with cell death [18]. These studies on DTI cannot be considered equally with pressure ulcers on the foot discussed in the present study because the malleolus and the heel do not have subcutaneous muscles. However, both superficial and deep tissue develop pressure ulcers by local stress and strain due to continuous pressure or/and shear forces [19]. Thus, deformations of the internal tissues induced by the proposed passive motion have a possibility to attenuate the impact of sustained tissue/cell deformation caused by prolonged shear load. Experiments using magnetic resonance imaging (MRI) and/or finite element models will be helpful to get better understanding.

 The current results indicate that a passive motion system for toe joints will be effective in preventing ulcer formation on the foot. Since the present study measured only blood flow, the effect of toe joints' passive motion toward tissue/cell deformation remains unclear. Further experiments are needed to reveal the detailed mechanism.

### 4.2. Appropriate Angle and Force Ranges in the Prevention System

The extension range of motion (ROM) has been reported to be 60 degrees at the first MTP joint, 40 degrees at the second through fifth MTP joints, and 0 degree at the first interphalangeal (IP) joint and the second through fifth proximal interphalangeal (PIP) and distal interphalangeal (DIP) joints [20]. Flexion and extension of participant's first through fifth joints were performed at one time in the experiment as mentioned previously. Using this procedure appears to lessen extension angle (Figure 4) when compared with ROM of the first MTP joint. On the other hand, the flexion ROM is 35 degrees at the first through fifth MTP joint and 50 degrees to 60 degrees at the first IP joint and the second through fifth PIP and DIP joints. The flexion angle was approximately the same as the ROM of the first MTP joint. We believe that the flexion motion was limited by the ROM of the first MTP joint in our experiment.

The results shown in Figure 4 can be used to determine the appropriate angle and force ranges for a system to prevent pressure ulcers. However, as it is previously mentioned, the ranges of force and angles measured during flexion and extension were differed largely among participants. Thus, a passive motion system for toe joints should have the ability to adapt the output force and angle ranges according to the individual needs of each users.

### 4.3. Evaluation of an MH Actuator for Future Work

On the basis of our results, a new passive motion system for the prevention of pressure ulcers may be designed. In this study, we suggest the use of an actuator employing an MH alloy (MH actuator) as an operating part of the prevention system. Although there are many types of actuators, such as hydraulic and pneumatic actuators and motors, an MH actuator offers the following advantages: high power-to-weight ratio, silent operation, zero vibration, and a mechanical buffering action resembling that of a cushion.

**Figure 5:**
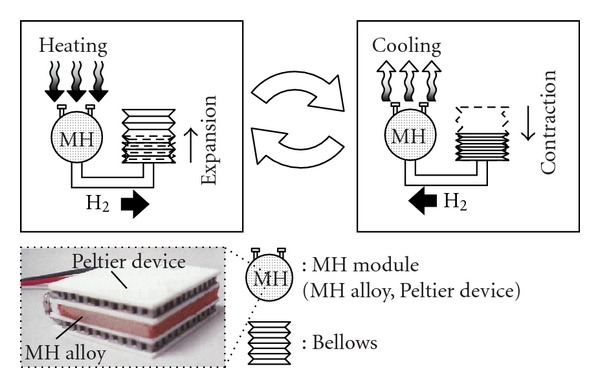
Operating principle of the MH actuator.

**Figure 6:**
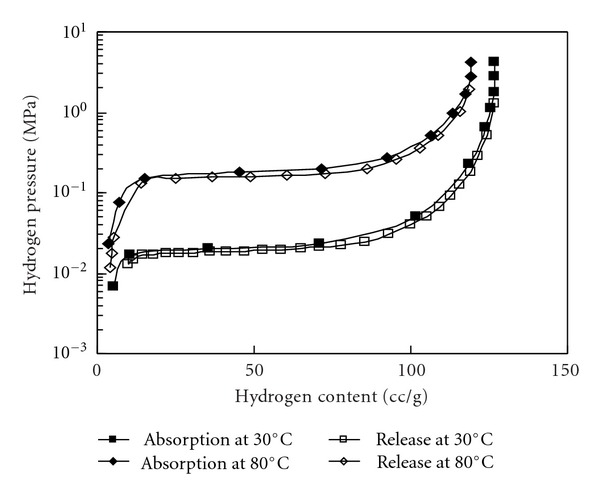
*P-C-T* diagram of the MH alloy (LaNi_5_).

 MH is an alloy able to reversibly absorb and release a considerable amount of hydrogen according to the following equation:

(1) $$\left(\frac{2}{x}\right)\text{M}+{\text{H}}_{2}⇆\left(\frac{2}{x}\right){\text{MH}}_{x}+Q,$$

where M denotes a hydrogen absorbing alloy, H_2_ denotes hydrogen, MH_*x*_ denotes the resulting metal hydride, and *Q* denotes the quantity of heat involved in the reaction, generally expressed as *Q* > 0 J/mol. The function of an MH actuator is shown in Figure 5. An MH alloy with Peltier devices as thermoelectric heating and cooling elements is sealed in a container (MH module). The MH actuator derives mechanical power by converting the internal hydrogen pressure changes of the MH module into the expansion and contraction movement of a bellows.  Figure 6 shows a *P-C-T* diagram (where *P* is hydrogen pressure, *C* is hydrogen content and *T* is temperature) of the MH alloy used in this study. According to Gibb's Law, the reaction will proceeds at a constant pressure (the plateau pressure) if the temperature is maintained at a constant level as shown in Figure 6.

**Figure 7:**
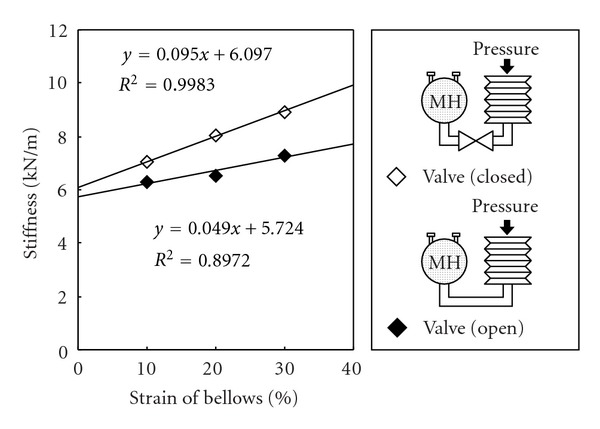
Stiffness of a bellows with and without an MH module.

An MH alloy can absorb hydrogen gas not only by cooling but also through the application of pressure.  Figure 7 shows changes in the stiffness of an MH actuator, with and without a closed valve between the MH module and the bellows, during a pressure test performed using a universal tester (STA-1225, ORIENTEC Co., Ltd.). The initial internal hydrogen pressure was 111 kPa. A bellows made from laminated aluminum film (CPP/Al/PET, 0.1 mm in thickness) was utilized as an end effector for the MH actuator (soft MH actuator) to ensure impermeability to hydrogen gas and user safety [21]. The bellows was 100 mm in diameter and weighed 40 g when configured with 19 active corrugations. The cross-sectional area of the bellows was 5000 mm^2^ and the stiffness was calculated using Hooke's law. As shown in Figure 7, the gradient of the stiffness became lower when a closed valve between the MH module and the bellows was removed. This effect was caused by the MH alloy as it absorbed hydrogen in response to an applied pressure on the surface of the bellows. It is considered that this characteristic may act as shock-absorbing function in the system when unexpectedly high force is applied to the MH actuator.

**Figure 8:**
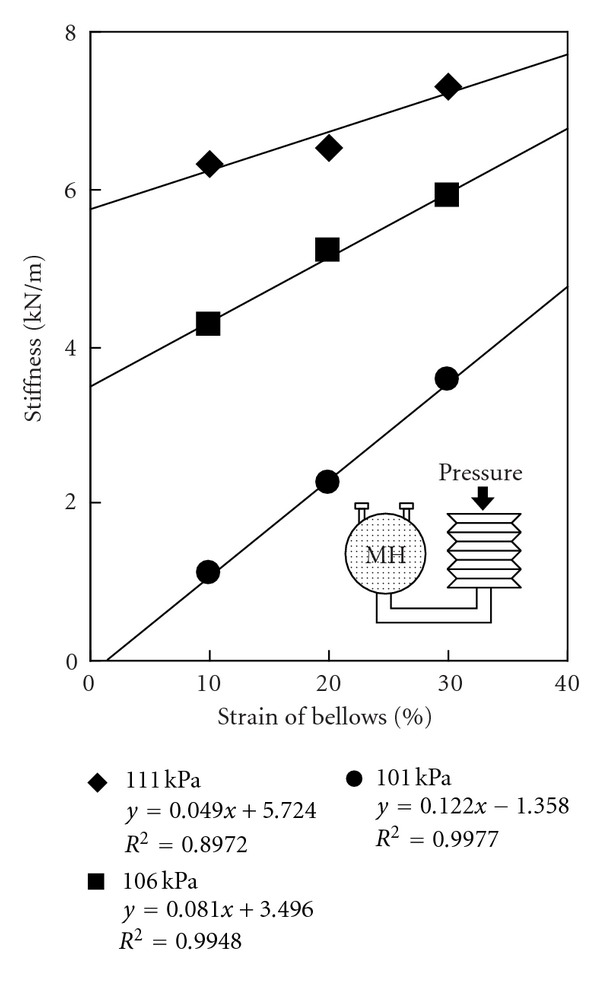
Stiffness of the soft MH actuator.

The change in stiffness of an MH actuator as a function of initial internal hydrogen pressures was also measured (Figure 8). Initial internal pressure of the MH actuator was 101 kPa, 106 kPa and 111 kPa, respectively. As shown in Figure 8, the stiffness and output force of the MH actuator can be controlled by adjusting the internal pressure. The desired output range of pressure is from 1.5 kPa to 4.0 kPa, as calculated from the results shown in Figure 4. This is an achievable range using the proposed actuator design.

**Figure 9:**
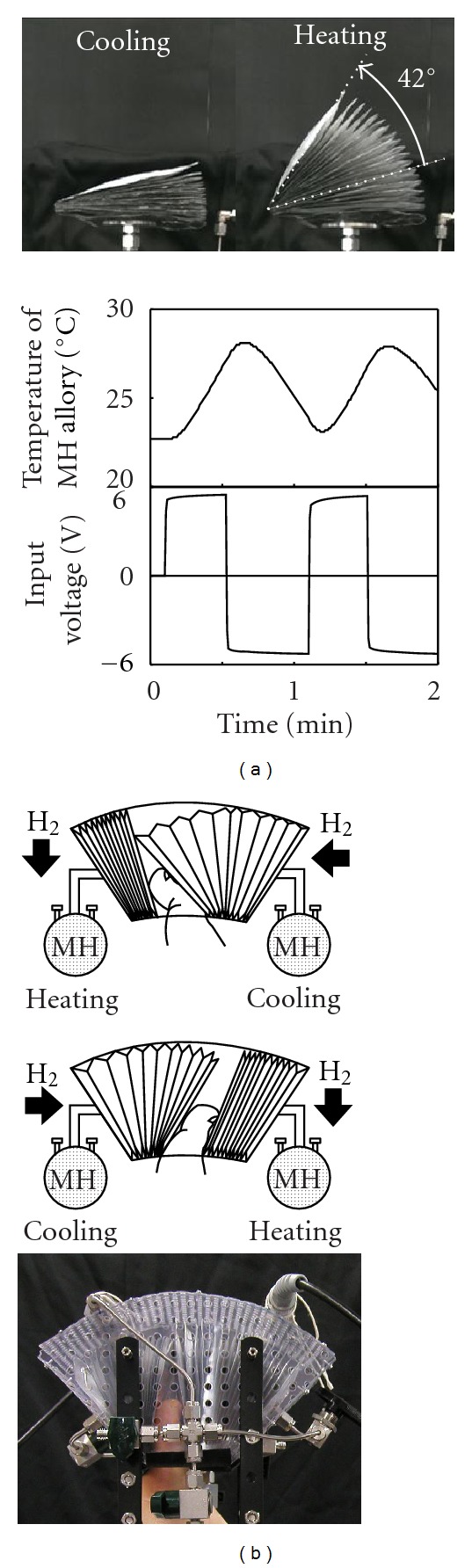
(a) Step response of the soft MH actuator, (b) antagonistic action of two soft MH actuators.

Figure 9(a) shows the step response of the soft MH actuator in response to an applied voltage of ±5 V. The bellows was pinned so that its hinge-bending action could be realized. The mean value of its maximum bending angle was 42 degrees. Comparing this result to the result shown in Figure 4, it is expected that flexion and extension of the toe can be realized by applying antagonistic actions of two bellows (Figure 9(b)).

 Based on the above results, we believe that the soft MH actuator is suitable as a part of a system for the passive motion of toe joints. Development of the system and its evaluation will be important future work.

## 5. Conclusions

Increases in blood flow at the superior surfaces of the first metatarsal head (*P* < 0.01, Wilcoxon test) and the external malleolus (*P* < 0.02, Wilcoxon test) were observed by the passive flexion and extension of toe joints. This result suggests the utility of a motion device for toe joints to prevent pressure ulcers. The flexion and extension angles and force of the toe joints were measured to determine appropriate specifications for the system. Force ranges differed significantly among participants, ranging from 8 N to 14 N for flexion and from 9 N to 20 N for extension. Similarly, the angles of the MTP joint differed from 24 degrees to 47 degrees in flexion and from 16 degrees to 40 degrees in extension. Based on these results, an actuator using an MH alloy was designed and evaluated to determine suitability for a prevention system for pressure ulcers.
